# The Role of Functional Polymorphisms in the Extracellular Matrix Modulation-Related Genes on Dupuytren’s Contracture

**DOI:** 10.3390/genes13050743

**Published:** 2022-04-23

**Authors:** Gediminas Samulenas, Ruta Insodaite, Edita Kunceviciene, Roberta Poceviciute, Lorena Masionyte, Urte Zitkeviciute, Loreta Pilipaityte, Alina Smalinskiene

**Affiliations:** 1 Department of Plastic and Reconstructive Surgery, Lithuanian University of Health Sciences, LT 50009 Kaunas, Lithuania; gediminas.samulenas@lsmu.lt (G.S.); loreta.pilipaityte@lsmu.lt (L.P.); 2 Institute of Biology Systems and Genetics Research, Lithuanian University of Health Sciences, LT 50103 Kaunas, Lithuania; edita.kunceviciene@lsmuni.lt (E.K.); roerta.poceviciute@lsmu.lt (R.P.); lorena.masionyte@lsmu.lt (L.M.); alina.smalinskiene@lsmuni.lt (A.S.); 3 Faculty of Medicine, Lithuanian University of Health Sciences, LT 44307 Kaunas, Lithuania; urte.zitkeviciute@stud.lsmu.lt

**Keywords:** Dupuytren’s contracture, single-nucleotide polymorphism, extracellular matrix, *MMP8*, *MMP14*, *CHST6*

## Abstract

(1) Background: genetic variations, localized in the functional regions of the extracellular matrix (ECM) modulation-related genes, may alter the transcription process and impact the Dupuytren’s contracture (DC). The present study investigated the association of single nucleotide polymorphisms (SNPs), localized in the functional regions of the *MMP8*, *MMP14*, and *CHST6* genes, with DC risk. (2) Methods: we enrolled 219 genomic DNA samples, which were extracted from 116 patients with DC and 103 healthy controls. Genotyping of selected SNPs was performed using TaqMan single nucleotide polymorphisms genotyping assay. Three polymorphisms (*MMP8* rs11225395, *MMP14* rs1042704, and *CHST6* rs977987) were analyzed. All studied SNPs were in Hardy–Weinberg equilibrium. (3) Results: significant associations of the studied SNPs with the previous onset of the disease were observed between the *CHST6* rs977987 minor T allele (*p* = 0.036) and the *MMP14* rs1042704 mutant AA genotype (*p* = 0.024). Significant associations with the previous onset of the disease were also observed with a positive family history of the DC (*p* = 0.035). Moreover, risk factor analysis revealed that a combination of major disease risk factors (smoking and manual labor) and the *MMP14* minor A allele increases the risk of DC development by fourteen times (*p* = 0.010). (4) Conclusions: our findings suggest that *CHST6* rs977987, *MMP14* rs1042704, and positive family history are associated with the previous onset of Dupuytren’s contracture. In addition, the combination of the *MMP14* minor A allele and additional risk factors increase the likelihood of the manifestation of the DC.

## 1. Introduction

Dupuytren’s contracture manifests as a chronic hand condition, causing fibroproliferative alterations in the structure of the palmar aponeurosis, resulting in progressive flexion contractures of finger joints [1,2,3]. Disease prevalence rates range from 0.6% to 31.6% in different population groups. Significantly higher rates of DC are observed among middle-aged or older males of Northern European descent [4,5]. Epidemiological studies have suggested a correlation with alcoholism, smoking, increasing age, male gender, diabetes, and epilepsy [6]. Also, genetic predisposition plays a major role in the development of this condition. Studies show that genetic factors, and especially heredity, account for 80% of the factors involved in causing this disease [7]. A genome-wide association study identified 26 risk alleles associated with DC [8,9]. Despite this, the pathogenesis of DC disease has not been fully explained.

In Luck classification, Dupuytren’s tissue has been reported to contain several histological zones [10]. The proliferative or active zone is characterized by the presence of a large number of (myo)fibroblasts and the development of a nodular lesion. The involution zone contains large amounts of collagens and myofibroblasts. Cells in the involution zone align themselves to lines of stress. The final stage or the residual zone leaves scar-like cord tissue. The growing nodules and the arrangement of newly formatted fibers entail tissue reorganization coupled with degradation of the surrounding ECM [11,12].

The major enzymes implicated in ECM degradation are considered matrix metalloproteinases (MMP) or matrixsins. MMPs are calcium-dependent, zinc-containing endopeptidases involved in tissue remodeling by interfering with the cell-cell and cell-extracellular matrix interactions [13]. In general, MMPs degrade different kinds of ECM components, including collagens, elastin, gelatin, matrix glycoproteins, proteoglycan, other ECM molecules, and soluble proteins [14]. Different MMPs (MMP2, MMP9, MMP14) and their tissue inhibitors (TIMP1 and TIMP2) expression were studied in DC tissue, but only a few have been prognostically significant [15,16]. Ulrich and coauthors show a significant correlation between collagenase *MMP2* expression changes and DC [12]. Additionally, some studies show that *MMP14* is over-expressed in DD nodules, and knockdown of *MMP14* in DC-derived cells reduced both contraction and *MMP2* activation in vitro [15,17]. Another potential biomarker for DC, due to its ability to degrade type I collagen three times more patently compared to other collagenases (MMP1, MMP13, and MMP18), could be MMP8 [18]. According to biochemical analysis, the DC cord demonstrated the abundance of type I collagen [19,20]. These findings can be explained by *MMP8* and other collagenases’ expression changes in DC pathogenesis; however, the association between these MMPs and this pathology is poorly investigated.

A genome-wide association study identifies another with ECM changes in DC-related gene carbohydrate sulfotransferase 6 (*CHST6*), which is involved in the biosynthesis of keratan sulfate [8]. Keratan sulfate is a carbohydrate moiety of the proteoglycans found especially in the corneal stroma, extracellular matrix, cartilage, and bone [21]. Studies showed that mutations in *CHST6* cause a keratan sulfate metabolism change, resulting in the deposition of an unsulfated proteoglycan, both within the intracellular and also in the extracellular space, and disturbance of the structural integrity of the tissues. Most studies analyzed the association between *CHST6* mutations, their caused expression changes, and macular corneal dystrophy, but to our knowledge, the relation between this gene and DC was analyzed in only one study [22].

Single nucleotide polymorphisms in the genes related to ECM changes may play a role in the development of DC. Three functional SNPs in *MMP14* (rs1042704), *MMP8* (rs11225395), and *CHST6* (rs977987) were selected. *MMP14* gene polymorphism rs1042704 results in missense mutation that changes both the sequence and the function of the *MMP14*. Studies have shown that the altered protein exhibited significantly less collagen degradation activity compared to the wild-type enzyme [23]. Another polymorphism, rs11225395, is localized in the *MMP8* gene promoter region causing changes in protein expression levels [24,25,26]. The third selected polymorphism is localized in the 3′-untranslated region of the *CHST6* gene and may cause protein expression changes due to modifications in the miRNA binding site [27]. Genome-wide association study showed possible links between selected *MMP14* and *CHST6* gene polymorphisms and DC [8]. However, to the best of our knowledge, there is no data in the literature about the possible links between *MMP8* gene polymorphisms and DC development.

Therefore, the present study aimed to investigate the contributions of *MMP8*, *MMP14*, and *CHST6* gene functional polymorphisms to the development of DC.

## 2. Materials and Methods

### 2.1. Ethics Statement

Permission to undertake the study was obtained from the Kaunas Regional Biomedical Research Ethics Committee (Number No. BE-2-21, 08 March 2019) and from the Department of Bioethics, LUHS (BEC-MF-63. 23 October 2017). Before the study, the procedure and purpose of the study were explained, and informed consent was obtained from all participants.

### 2.2. Study Population

We collected peripheral blood samples of 219 day-case patients (116 DC patients and 103 healthy controls), receiving surgery in the Department of Plastic and Reconstructive Surgery, Hospital of LUHS (Table 1). The DC group included patients with diagnosis of Dupuytren’s contracture. The criteria for the control group were no clinical signs or previous diagnosis/treatment of DC or stenosing tenosynovitis and no family history of Dupuytren’s contracture. Patients younger than 30 years were excluded from the study. All patients were indigenous Lithuanians. Data on patients’ general health and concomitant conditions were acquired from clinical examination and medical records.

### 2.3. Candidate Polymorphisms

The genes and polymorphisms known to participate in ECM modulation were selected. The selection criteria included: (i) functional SNPs in *MMP8*, *MMP14*, and *CHST6* genes; (ii) SNPs relevant to outcomes in other settings; and (iii) SNPs with minor allele frequency greater than 10% in the study population. We selected three functional SNPs: the *MMP14* gene rs1042704; the *MMP8* gene rs11225395; and the *CHST6* gene rs977987.

### 2.4. DNA Extraction and Genotyping

Peripheral blood samples were collected from each individual in ethylenediaminetetraacetic (EDTA) tubes for DNA extraction. DNA was extracted from leukocytes using a reagent kit (NucleoSpin Blood L Kit; Macherey & Nagel, Düren, Germany) according to the manufacturer’s recommendations.

Four SNPs of the *MMP8*, *CHST6*, and *MMP14* genes were assessed using commercial genotyping kits (C_175744753_10, C___2848998_10, C__11436237_20, Applied Biosystems, Foster City, CA, USA). The Applied Biosystems 7900HT Real-Time Polymerase Chain Reaction System (Applied Biosystems, Foster City, CA, USA) was used for detecting the SNPs. The cycling program started with heating for 10 min at 95 °C, followed by 40 cycles of 15 s at 95 °C and 1 min at 60 °C. Allelic discrimination was performed using the SDS 2.3 software (Applied Biosystems, Foster City, CA, USA). For negative control, nuclease-free ddH_2_O was used, while for positive control, the DNA of the known genotype was used. Each sample genotyping was repeated twice for accuracy.

### 2.5. Statistical Analysis

For each SNP, a Hardy–Weinberg equilibrium was assessed by using Pearson’s chi-square and Fisher’s exact test. Age between groups was compared using Student’s *t*-test. Allele and genotype frequencies between DC and control groups were compared by Pearson’s chi-square test. The association between the *MMP8*, *CHST6*, and *MMP14* polymorphisms and DC was estimated by computing the odds ratios (ORs) and then 95% confidence intervals (CI) from logistic regression in genotype and allele models. The ORs and CI from Pearson’s chi-square test were calculated to evaluate the effects of risk factors on DC. Results were statistically significant when *p* was less than 0.05. Statistical analysis was performed using SPSS v20.0 software (Released 2011; IBM Corp, Armonk, NY, USA).

## 3. Results

### 3.1. Sample Characteristics

A total of 116 patients with DC were included in the current analysis. All 103 persons from the control group were also included. The DC patients and reference groups were matched by age (*p* > 0.05). Detailed group demographics and characteristics are summarized in Table 1.

All subjects were genotyped for a panel of three SNPs: the *MMP8* gene rs11225395; the *CHST6* rs977987; and the *MMP14* rs1042704. The genotypes were found to be in Hardy–Weinberg equilibrium in the four SNPs.

### 3.2. Case-Control Analysis

The distribution of *MMP8*, *CHST6*, and *MMP14* genotypes and alleles in DC and control groups are presented in Table 2. No statistically significant results were observed in genotype and allele distribution between patients and control groups. Statistically significant results were also not obtained in the logistic regression analysis where the effect of *MMP8*, *CHST6*, and *MMP14* SNPs on DC development was analyzed (Table 3).

To analyze the associations of *MMP8*, *CHST6*, and *MMP14* polymorphisms with the previous onset of the disease, we divided the DC patients into groups (>56 and ≤56) according to the age when the first symptoms appeared. The analysis results on the association between analyzed SNPs and the previous onset of the DC are shown in Table 4 and Table 5. A significant link between the *CHST6* rs977987 mutant TT genotype and the previous onset of the DC (OR 1.691; 95% CI: 1.465–3.737; *p* = 0.044) was revealed. The allelic model showed that the major allele G of this SNP is associated with the subsequent onset of the disease (OR 0.591; 95% CI: 0.268–0.831; *p* = 0.029), in contrast to minor T allele (OR 1.404; 95% CI: 1.571–3.453; *p* = 0.036). Additionally, *MMP14* polymorphism rs1042704 in the genotype model was linked to the previous onset of the DC. Specifically, 16.8% of DC patients carrying the *MMP14* rs1042704 AA genotype first symptoms of DC were observed at less than 56 years of age, compared to 3.2% of noncarriers (OR 2.160; 95% CI: 2.160–7.824; *p* = 0.024).

### 3.3. The Previous Onset of the Disease and Positive Family History

The relationship between the incidence of DC cases among relatives of DC patients and the previous onset of the disease was evaluated. In the DC group, 33 out of 116 cases were hereditary DCs. We found that a family history of DCs significantly associated with the previous onset of the disease (OR: 2439; 95% CI: 1.055–5.642; *p* = 0.035) (Table 6). The relationships between our examined polymorphisms and heritability of DC did not exhibit statistically significant differences.

### 3.4. Risk Factor Analysis

Risk analysis was also conducted to examine the impact of anthropometric lifestyle factors and their combination with analyzed gene polymorphisms on Dupuytren’s contracture (Table 7). Compared with non-smokers, persons who are smokers were 2.615 times (95% CI: 1.114–3.898; *p* = 0.020) more likely to develop DC. Manual labor was associated with 2.615 times increased risk of DC. Compared with non-smokers and non-manual laborers, persons who are smokers and manual laborers were 13.174 times (95% CI: 3.029–57.290; *p* < 0.001) more likely to develop Dupuytren’s contracture. Finally, the most high-risk factor (OR: 14.00) of DC development was for persons who have a combination of smoking, manual labor, and *MMP14* rs1042704 minor A allele (95% CI: 1.807–108.450; *p* = 0.010).

## 4. Discussion

A genome-wide association study (GWAS) has identified five harbor loci genes associated with DC that are known to interact with and modulate the ECM. The researchers report that not all the links between genes and DC identified by GWAS may be clear and, therefore, require experimental verification [8]. We investigated two SNPs of the rs1042704 in *MMP14* and the rs977987 in the *CHST6* genes from GWAS and one SNP of rs11225395 in *MMP8*, which were of interest primarily because of the lack of literature on the *MMP8* gene and DC.

Many studies are being conducted on the balance of MMPs and TIMPs inhibitors for diseases involving tissue destruction. It is already known that the high expression of MMPs can cause increased synthesis and deposition of collagen, leading to palmar fibromatosis [12]. Previous studies have shown that the *MMP14* gene polymorphism rs1042704 causes a missense mutation that alters the *MMP14* sequence. Altered expression of the *MMP14* gene alters a protein with lower collagen degradation activity [23]. Our study also suggests that 2.2 times more frequent occurrence of the A minor allele of the SNP rs1042704 in the *MMP14* gene in the DC group may be related to an unsuccessful attempt to reduce collagen deposition.

The genome-wide association study has shown possible links between the selected *MMP14* and *CHST6* gene polymorphisms and DC [8]. In the current study, subjects with the mutant TT genotype of SNP rs977987 in the *CHST6* gene were 1.7 times more likely to develop DC. Therefore, our study complements the GWA study and confirms that *MMP14* and *CHST6* are significant for DC development.

The third polymorphism we studied, SNP rs11225395 in *MMP8*, is localized in the promoter region of the *MMP8* gene, and changes in the sequence of the *MMP8* gene are known to cause changes in protein expression [24]. However, our studies did not show significant associations between the SNP rs11225395 *MMP8* gene and DC. No further publications related to *MMP8* gene studies and DC were found.

We did not find any significant interfaces between SNP rs11225395 in the *MMP8* gene and DC. The view of other researchers that not all MMPs show signs of tissue-degrading enzymes should probably be accepted. Other publications’ description that MMP8 has more anti-inflammatory effects in the treatment of anti-inflammatory cytokines and chemokines should probably be shared [28,29].

In this study, we examined several potential DC risk factors, such as age, gender, smoking, manual labor, and their combinations with SNP polymorphisms of the *MMP8*, *MMP14*, and *CHST6* genes.

We found that SNP rs1042704 AA in *MMP14* and *CHST6* rs977987 mutant TT genotype were significantly associated with the early onset of DC.

There is another study that proves that older age is not associated with the efficacy of MMP14. Only a few MMPs have been identified in this study whose activity is increased in long-lived individuals compared to younger ones, one of them being MMP2 [30].

The occurrence of DC is most common in populations of Northern European/Scandinavian origin and uncommon in populations in Southern Europe and South America [31]. Men suffer from DC at a 2:1 ratio compared to women, and men also suffer worse [32]. Our sample of subjects who reported DC onset also had a higher proportion of men (83.6%) than women (16.4%). This suggests that the male gender is a very important risk factor for the onset of DC.

Cigarette smoking is statistically associated with Dupuytren’s disease [32]. We found that smokers were statistically 2.084 times more likely to develop DC than non-smokers. Other researchers have also found that DC is 1.3 to 2.8 times more common in smokers than in non-smokers [33,34].

Although DC is not considered an occupational disease, in a study of workers, 3.76% were affected by DC, especially younger workers. It is also estimated that it takes 10 years of hard manual labor to develop [35]. Our study revealed that manual laborers are 2.6 times more likely to develop DC than non-manual laborers. We also found that a combination of strong risk factors, smoking and manual labor, resulted in a 13.2-fold increase in DC incidence compared to non-smokers and non-manual laborers. The combination of these risk factors has not been described in previous publications. However, our study revealed that these risk factors significantly determine the occurrence of DC. Finally, we found an even greater propensity for DC development when smoking, manual labor, and the *MMP14* rs1042704 A allele interact together. The possibility of DC development increased 14 times. Riesmeijer et al. conducted an analysis of risk alleles in DCs with SNPs in African, East Asian, European, Hispanic, and South Asian populations and found that the frequency of alleles varies across populations [36]. Therefore, DC genetic testing in different populations can help predict the onset of the disease and pre-select preventive measures, such as risk factor reduction (smoking, manual labor), which, as our research shows, increases the occurrence of DC by several times.

## 5. Conclusions

Single nucleotide polymorphisms in *CHST6* rs977987, *MMP14* rs1042704, and positive family history are associated with the previous onset of Dupuytren’s contracture: *CHST6* rs977987 minor allele T increase the chances by 1.404-fold, *MMP14* rs1042704 mutant genotype AA—2.160-fold, and positive family history by two and a half times. Our study also showed that a combination of the *MMP14* minor A allele and additional risk factors such as smoking and manual labor appears to increase the likelihood of the manifestation of DC by fourteen times.

## Figures and Tables

**Table 1:** Characteristics of study groups.

Characteristic.	DC (*n* = 116)	Control (*n* = 103)	*p*-Value
Age (years)	60.24 (SD 10.665)	58.85 (SD 14.012)	0.415 ^1^
Gender:			<0.001 ^2^
Male	97 (83.6%)	65 (63.1%)	
Female	19 (16.4%)	38 (36.9%)	

^1^ Student’s *t*-test, for independent samples. ^2^ Significant differences, chi-squared comparison of patients’ gender between study groups. DC: Dupuytren’s contracture. SD: standard deviation.

**Table 2:** Distribution of genotypes and allele frequencies for polymorphisms at *MMP8*, *CHST6*, and *MMP14* genes in Dupuytren’s contracture (*n* = 116) and control (*n* = 103) groups.

Gene	SNP; Position	Group	Genotype Frequency			*p*-Value	MAF	*p*-Value
*MMP8*	rs11225395		AA	AG	GG	0.496	A	0.477
	G > A	DC	25 (21.6%)	60 (51.7%)	31 (26.7%)		47.4	
	Chr.11:102725749	Control	19 (18.4%)	49 (47.6%)	35 (34.0%)		42.2	
*CHST6*	rs977987		TT	GT	GG	0.708	T	0.568
	G > T	DC	25 (21.6%)	55 (47.4%)	36 (31.0%)		45.3	
	Chr.16:75472695	Control	19 (18.4%)	47 (45.6%)	37 (35.9%)		41.3	
*MMP14*	rs1042704		AA	AG	GG	0.422	A	0.635
	G > A	DC	11 (9.5%)	46 (39.7%)	59 (50.9%)		29.3	
	Chr.14:22843385	Control	5 (4.9%)	43 (41.7%)	55 (53.4%)		25.7	

DC: Dupuytren’s contracture. MAF: minor allele frequency.

**Table 3:** Association between *MMP8*, *CHST6*, and *MMP14* polymorphisms in allele and genotype models and Dupuytren’s contracture development.

Gene	SNP	Model	OR	95% CI	*p*-Value
*MMP8*	rs11225395 G > A	Genotype model:			
		GG vs. AG and AA	1.411	0.791–2.518	0.244
		AA vs. AG and GG	0.823	0.423–1.603	0.567
		AG vs. GG and AA	0.847	0.498–1.441	0.540
		Allelic model:			
		G carrier vs. G noncarrier	0.709	0.397–1.264	0.244
		A carrier vs. A noncarrier	1.215	0.624–2.365	0.567
*CHST6*	rs977987 G > T	Genotype model:			
		GG vs. TG and TT	0.825	0.423–1.603	0.567
		TT vs. TG and GG	1.246	0.710–2.187	0.444
		TG vs. GG and TT	0.931	0.547–1.585	0.792
		Allelic model:			
		G carrier vs. G noncarrier	0.803	0.457–1.409	0.444
		T carrier vs. T noncarrier	1.215	0.625–2.365	0.567
*MMP14*	rs1042704 G > A	Genotype model:			
		GG vs. AG and AA	1.107	0.651–1.883	0.708
		AA vs. AG and GG	0.487	0.163–1.452	0.197
		AG vs. GG and AA	1.091	0.635–1.872	0.753
		Allelic model:			
		G carrier vs. G noncarrier	0.903	0.531–1.537	0.708
		A carrier vs. A noncarrier	2.053	0.689–6.122	0.197

OR: Odds ratio. CI: Confidence intervals.

**Table 4:** Distribution of genotype and allele frequencies for polymorphisms at *MMP8*, *CHST6*, and *MMP14* genes in Dupuytren’s contracture patients according to the age when first symptoms appeared.

Gene	SNP	Age group	Genotypes			*p*-Value
*MMP8*	rs11225395		AA	AG	GG	0.834
		≤56	12 (22.2%)	29 (53.7%)	13 (24.1%)	
		>57	13 (21.0%)	31 (50.0%)	18 (29.0%)	
*CHST6*	rs977987		AA	AG	GG	0.024 ^1^
		≤56	24 (44.4%)	22 (40.7%)	8 (14.8%)	
		>57	12 (19.3%)	33 (53.2%)	17 (27.4%)	
*MMP14*	rs1042704		AA	AG	GG	0.040 ^1^
		≤56	9 (16.8%)	19 (35.1%)	26 (48.1%)	
		>57	2 (3.2%)	27 (43.5%)	33 (53.3%)	

^1^ Significant difference.

**Table 5:** The associations of *CHST6* and *MMP14* polymorphisms with the previous onset of the Dupuytren’s contracture.

Gene	SNP	Model	OR	95% CI	*p*-Value
*CHST6*	rs977987	Genotype model:			
		GG vs. GT and TT	0.712	0.290–1.751	0.459
		TT vs. GT and GG	1.691	1.465–3.737	0.044 ^1^
		GT vs. TT and GG	0.800	0.385–1.664	0.550
		Allelic model:			
		G carrier vs. G noncarrier	0.591	0.268–0.831	0.029 ^1^
		T carrier vs. T noncarrier	1.404	1.571–3.453	0.036 ^1^
*MMP14*	rs1042704	Genotype model:			
		GG vs. GA and AA	1.077	0.519–2.235	0.842
		AA vs. GA and GG	2.160	2.160–7.824	0.024 ^1^
		GA vs. GG and AA	0.704	0.332–1.491	0.359
		Allelic model:			
		G carrier vs. G noncarrier	0.463	0.278–0.678	0.024 ^1^
		A carrier vs. A noncarrier	0.929	0.448–1.927	0.842

^1^ Significant difference. OR: Odds ratio. CI: Confidence intervals.

**Table 6:** The association between the previous onset of the Dupuytren’s contracture and positive family history of the disease.

Age Group	Family History in DC		OR	95 % CI	*p*-Value
	Positive (*n* = 33)	Negative (*n* = 83)			
≤56 >57	21 (38.9%) 12 (19.4%)	33 (61.1%) 50 (80.6%)	2439	1.055–5.642	0.035 ^1^

^1^ Significant difference. OR: Odds ratio. CI: Confidence intervals. DC: Dupuytren’s contracture.

**Table 7:** The odds ratio for risk factors in Dupuytren’s contracture.

Parameters	Reference Values	Odds Ratio	95% CI	*p*-Value
Smoking	Non-smoking	2.084	1.114–3.898	0.020 ^1^
Manual labor	Non-manual labor	2.615	1.482–4.615	0.001 ^1^
Smoking + manual labor	Non-smoking + non-manual labor	13.174	3.029–57.290	<0.001 ^1^
*MMP14* rs1042704 A allele + smoking + manual labor	*MMP14* rs1042704 G allele + non-smoking + non-manual labor	14.000	1.807–108.450	0.010 ^1^

^1^ Significant difference. OR: Odds ratio. CI: Confidence intervals.

## Data Availability

Data supporting the reported results are available directly from the main author upon request.
